# Supplementary material for: Isoxanthohumol improves obesity and glucose metabolism via inhibiting intestinal lipid absorption with a bloom of Akkermansia muciniphila in mice
Source: Mol Metab. 2023 Sep 12;77:101797. doi: 10.1016/j.molmet.2023.101797 (PMC10539672; doi:10.1016/j.molmet.2023.101797)
Supplement: Multimedia component 3 [file mmc3.pptx]

## Slide 1
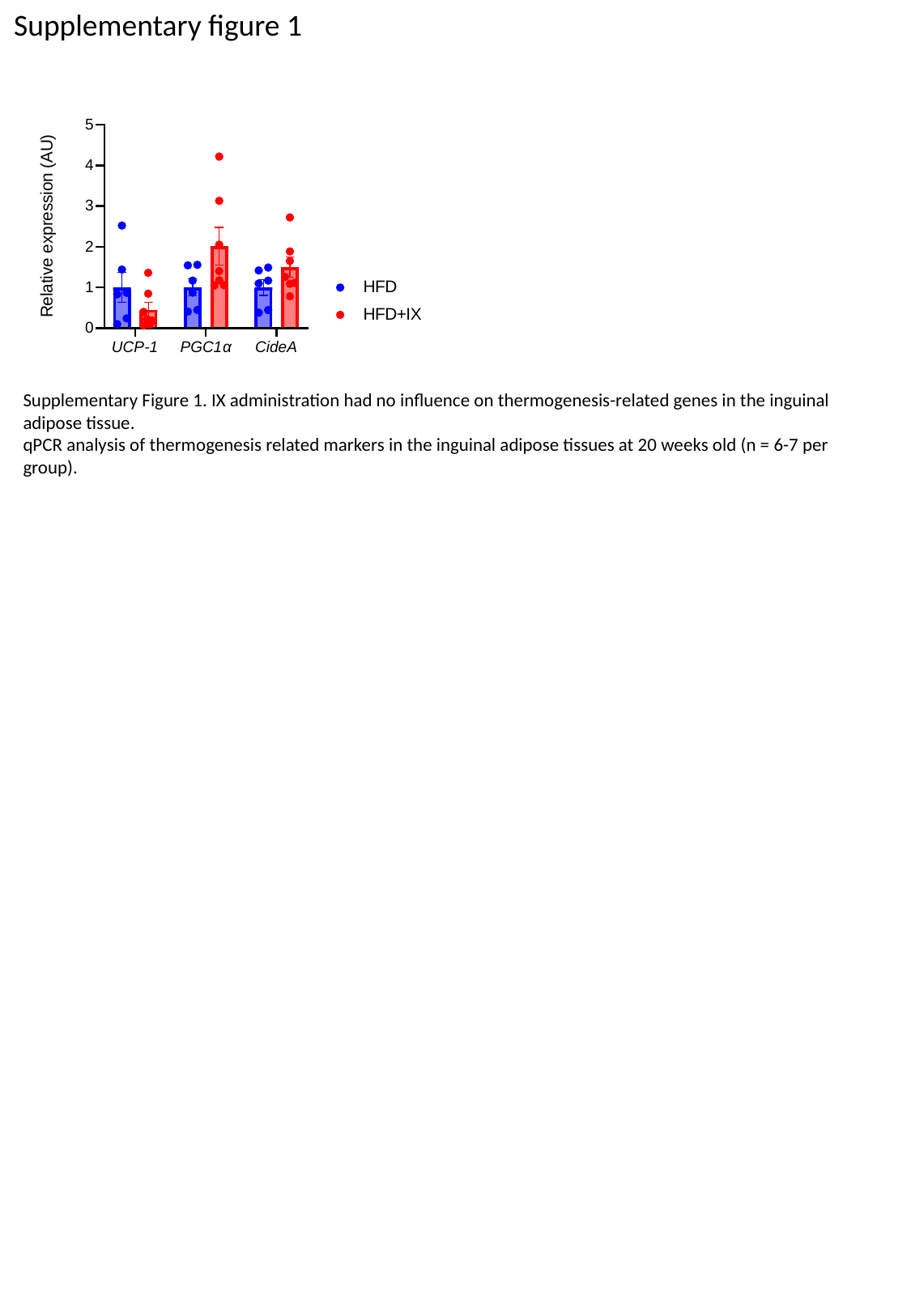

Supplementary figure 1
Supplementary Figure 1. IX administration had no influence on thermogenesis-related genes in the inguinal adipose tissue.
qPCR analysis of thermogenesis related markers in the inguinal adipose tissues at 20 weeks old (n = 6-7 per group).

## Slide 2
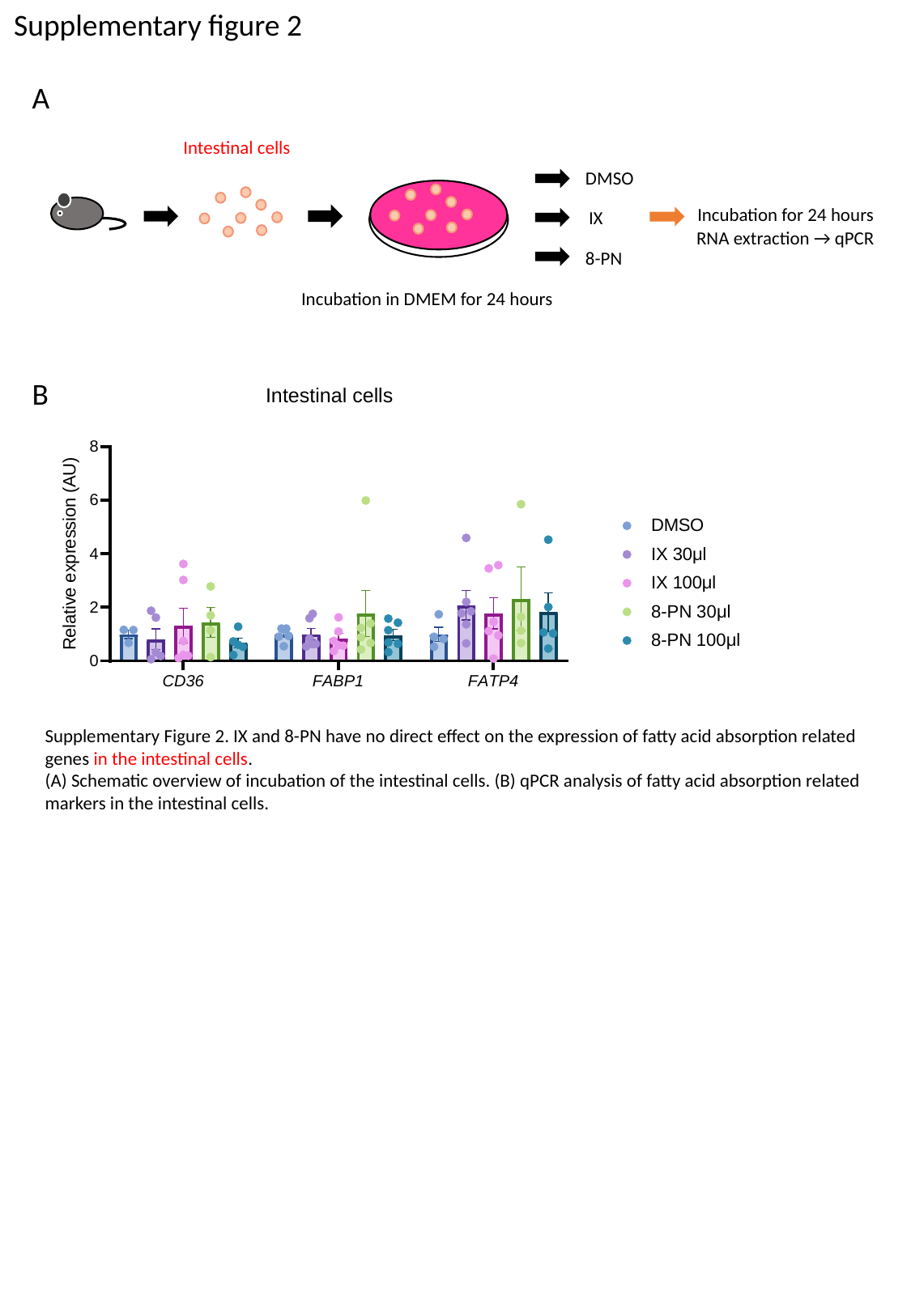

Supplementary figure 2
A
Intestinal cells
DMSO
Incubation for 24 hours
IX
RNA extraction → qPCR
8-PN
Incubation in DMEM for 24 hours
B
Intestinal cells
Supplementary Figure 2. IX and 8-PN have no direct effect on the expression of fatty acid absorption related genes in the intestinal cells.
(A) Schematic overview of incubation of the intestinal cells. (B) qPCR analysis of fatty acid absorption related markers in the intestinal cells.

## Slide 3
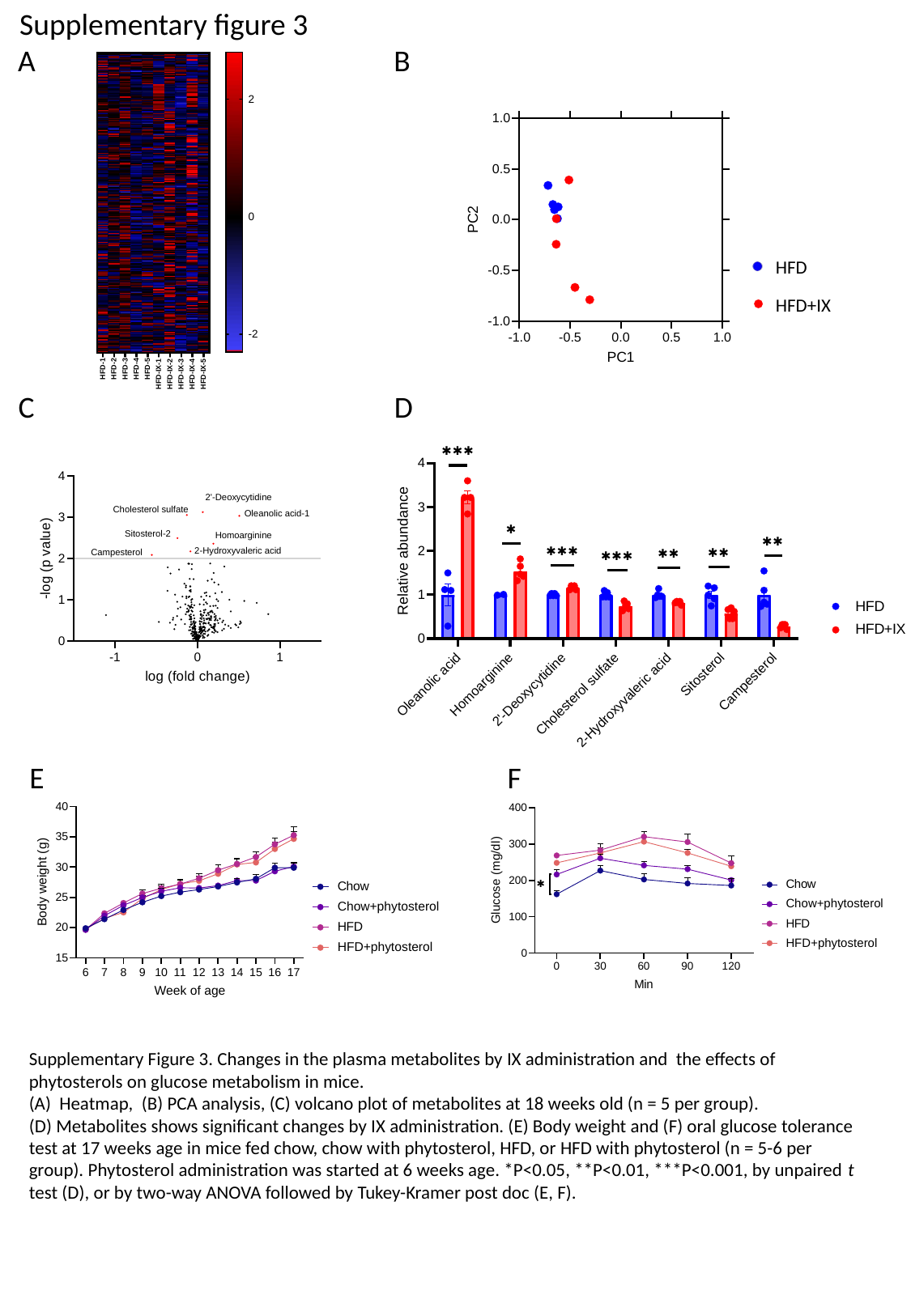

Supplementary figure 3
A
B
HFD
HFD+IX
C
D
E
F
Supplementary Figure 3. Changes in the plasma metabolites by IX administration and the effects of phytosterols on glucose metabolism in mice.
Heatmap, (B) PCA analysis, (C) volcano plot of metabolites at 18 weeks old (n = 5 per group).
(D) Metabolites shows significant changes by IX administration. (E) Body weight and (F) oral glucose tolerance test at 17 weeks age in mice fed chow, chow with phytosterol, HFD, or HFD with phytosterol (n = 5-6 per group). Phytosterol administration was started at 6 weeks age. *P<0.05, **P<0.01, ***P<0.001, by unpaired t test (D), or by two-way ANOVA followed by Tukey-Kramer post doc (E, F).

## Slide 4
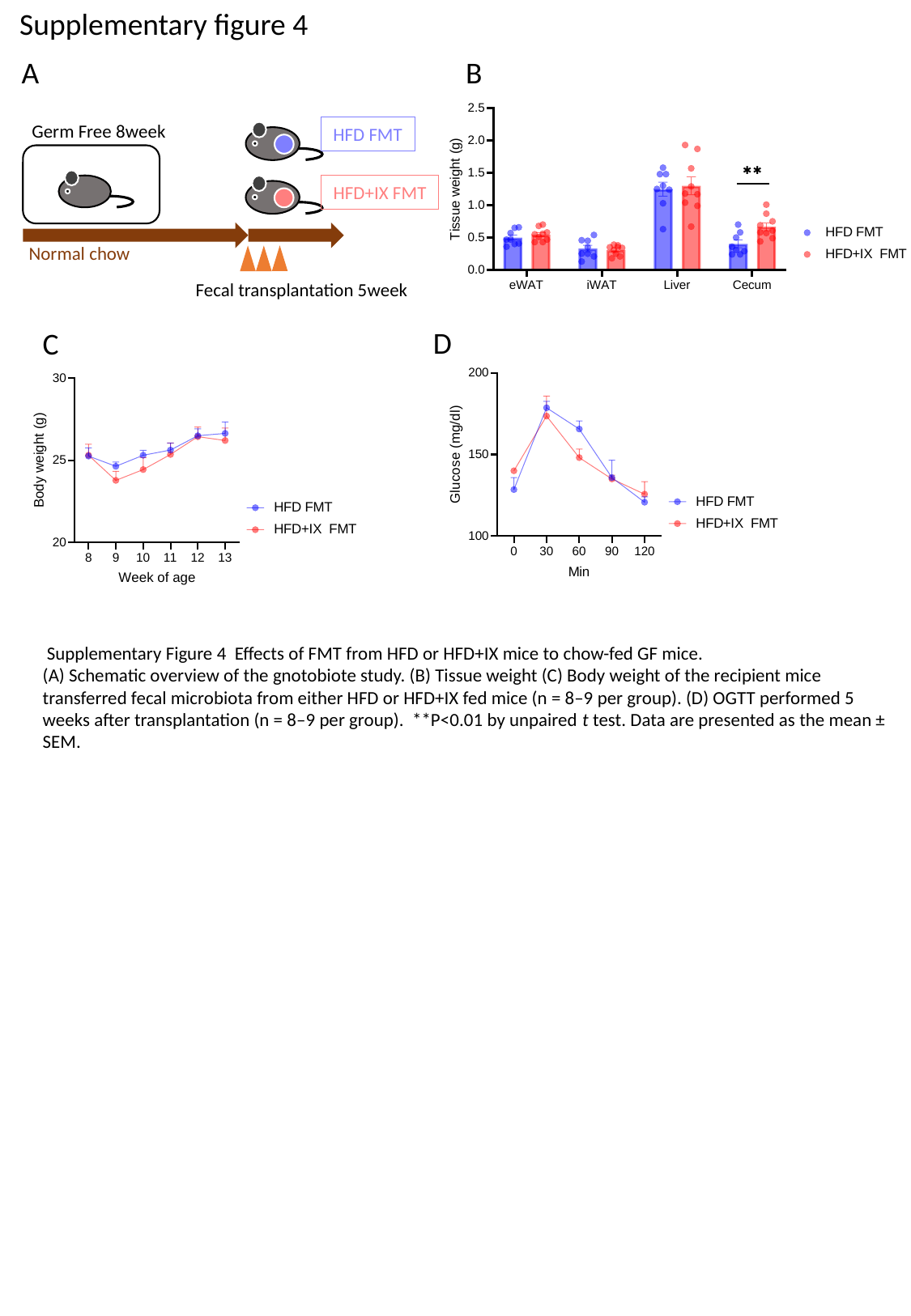

Supplementary figure 4
A
B
Germ Free 8week
HFD FMT
HFD+IX FMT
Normal chow
Fecal transplantation 5week
D
C
 Supplementary Figure 4 Effects of FMT from HFD or HFD+IX mice to chow-fed GF mice.
(A) Schematic overview of the gnotobiote study. (B) Tissue weight (C) Body weight of the recipient mice transferred fecal microbiota from either HFD or HFD+IX fed mice (n = 8–9 per group). (D) OGTT performed 5 weeks after transplantation (n = 8–9 per group). **P<0.01 by unpaired t test. Data are presented as the mean ± SEM.

## Slide 5
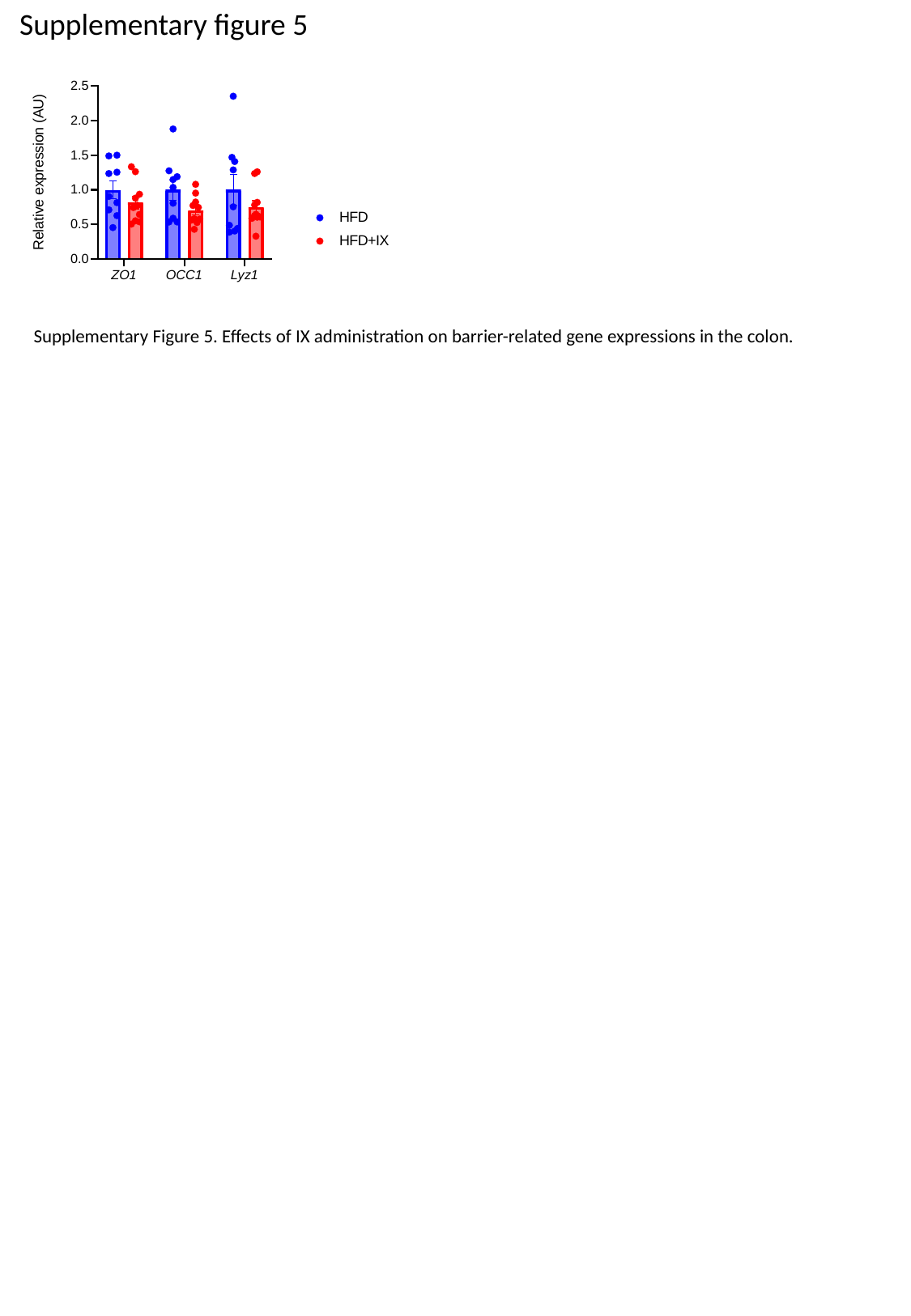

Supplementary figure 5
 Supplementary Figure 5. Effects of IX administration on barrier-related gene expressions in the colon.
